# Neurochemical Monitoring of Traumatic Brain Injury by the Combined Analysis of Plasma Beta-Synuclein, NfL, and GFAP in Polytraumatized Patients

**DOI:** 10.3390/ijms23179639

**Published:** 2022-08-25

**Authors:** Rebecca Halbgebauer, Steffen Halbgebauer, Patrick Oeckl, Petra Steinacker, Eberhard Weihe, Martin K.-H. Schafer, Francesco Roselli, Florian Gebhard, Markus Huber-Lang, Markus Otto

**Affiliations:** 1 Institute of Clinical and Experimental Trauma Immunology, Ulm University Hospital, Helmholtzstr. 8/1, 89081 Ulm, Germany; 2 Department of Neurology, Ulm University Hospital, Oberer Eselsberg 45, 89081 Ulm, Germany; 3 Deutsches Zentrum für Neurodegenerative Erkrankungen (DZNE e.V.), 89081 Ulm, Germany; 4 Department of Neurology, Halle University Hospital, Martin Luther University Halle-Wittenberg, Ernst-Grube Strasse 49, 06120 Halle (Saale), Germany; 5 Institute of Anatomy and Cell Biology, Philips-University Marburg, Robert-Koch-Straße 8, 35037 Marburg, Germany; 6 Center for Mind, Brain and Behavior, Philipps-University Marburg, Hans-Meerwein-Str. 6, 35032 Marburg, Germany; 7 Department of Orthopedic Trauma, Hand, and Reconstructive Surgery, University Hospital Ulm, Albert-Einstein-Allee 23, 89081 Ulm, Germany

**Keywords:** traumatic brain injury, multiple trauma, biomarkers in critical care, routine diagnostic tests

## Abstract

Traumatic brain injury (TBI) represents a major determining factor of outcome in severely injured patients. However, reliable brain-damage-monitoring markers are still missing. We therefore assessed brain-specific beta-synuclein as a novel blood biomarker of synaptic damage and measured the benchmarks neurofilament light chain (NfL), as a neuroaxonal injury marker, and glial fibrillary acidic protein (GFAP), as an astroglial injury marker, in patients after polytrauma with and without TBI. Compared to healthy volunteers, plasma NfL, beta-synuclein, and GFAP were significantly increased after polytrauma. The markers demonstrated highly distinct time courses, with beta-synuclein and GFAP peaking early and NfL concentrations gradually elevating during the 10-day observation period. Correlation analyses revealed a distinct influence of the extent of extracranial hemorrhage and the severity of head injury on biomarker concentrations. A combined analysis of beta-synuclein and GFAP effectively discriminated between polytrauma patients with and without TBI, despite the comparable severity of injury. Furthermore, we found a good predictive performance for fatal outcome by employing the initial plasma concentrations of NfL, beta-synuclein, and GFAP. Our findings suggest a high diagnostic value of neuronal injury markers reflecting distinct aspects of neuronal injury for the diagnosis of TBI in the complex setting of polytrauma, especially in clinical surroundings with limited imaging opportunities.

## 1. Introduction

Physical trauma is a major cause of hospital admission. Despite modern diagnostic and therapeutic strategies, trauma remains accountable for more than one in ten deaths worldwide [1]. Polytrauma is defined as multiple severe injuries where at least one injury or their combination is considered life-threatening [2]. The brain is the most frequently affected body region in traumatized patients, with head injuries affecting 46% of all trauma patients [3,4]. In this context, traumatic brain injury (TBI) represents a major risk factor for fatal outcomes [5]. Thus, novel, clinically valid, and reliable neurological markers for the longitudinal quantification of brain damage after severe injury with low invasiveness and rapid timing are needed [6]. In this regard, novel plasma synaptic biomarkers are urgently needed. The presynaptic protein beta-synuclein is almost exclusively expressed in the brain and has been shown to be highly elevated in early and advanced Alzheimer’s disease, Creutzfeldt–Jakob disease and Down syndrome [7,8,9,10]. Furthermore, we recently established highly sensitive immunoassay-based beta-synuclein detection in blood [11]. These data suggest beta-synuclein is released into the bloodstream as a consequence of synaptic structure degradation, accentuating its potential usefulness as a sensitive synaptic damage marker. Neurofilament light chain (NfL) protein, as a part of the cytoskeletal structure of neurons, is widely accepted as a neuroaxonal injury marker in the cerebrospinal fluid (CSF) and blood in various neurodegenerative diseases [12], with higher blood levels in relation to clinical outcomes after mild and severe TBI [13,14]. Glial fibrillary acidic protein (GFAP), as an astroglial injury marker, is detectable in the blood early after TBI [15,16], and high GFAP levels are associated with unfavorable outcomes [17,18].

Here, we hypothesized that the combined measurement of specific neurological damage markers is a suitable tool to detect TBI in the complex condition of multiple injury, even in clinical settings with limited (repetitive) imaging possibilities, and that distinct structural neuronal damage can be assessed employing synaptic, axonal, and glial marker proteins. In this regard, we also addressed gaps in the knowledge regarding axonal and astroglial injury in the time course after severe injury and their relation to clinical parameters of inflammation and shock.

## 2. Results

### 2.1. Clinical and Demographic Features

The clinical and demographic parameters are summarized in Table 1. There were no significant differences in the age and sex of the healthy controls and the trauma group. Age did not correlate with beta-synuclein, NfL, or GFAP in the control or the patient cohort.

### 2.2. Blood Neurochemical Marker Levels in the Time Course after Severe Trauma

**Figure 1:**
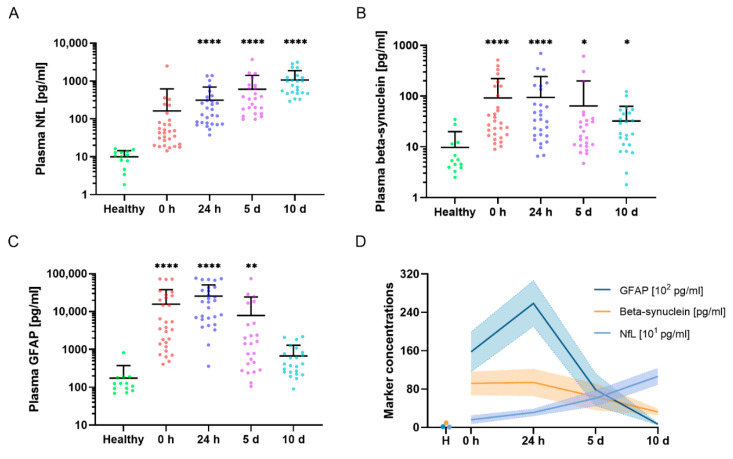
Systemic marker concentrations in the time course after severe injury. (**A**–**C**) Plasma NfL (**A**), beta-synuclein (**B**), and GFAP (**C**) concentrations in the time course after severe trauma compared to levels in healthy volunteers. Scatter plots are displayed for the individual sampling dates. The mean concentrations + SD are illustrated in (**A**–**C**). Groups were compared by Kruskal–Wallis test and Dunn’s post hoc test. **** *p* < 0.0001, ** *p* < 0.01, * *p* < 0.05 vs. healthy volunteers. (**D**) Time course of the observed marker concentrations in polytraumatized patients and healthy volunteers; since plasma concentrations differed by two decimal potencies, units were adjusted to align time courses in one graph; please see legend for respective units. Data are shown as means (solid lines) ± SEM (shaded zones). N = 13 (healthy), n = 29 (0 h), n = 28 (24 h), n = 24 (5 d), n = 23 (10 d) for (**A**–**D**). GFAP, glial fibrillary acidic protein; H, healthy volunteers; NfL, neurofilament light chain; SD, standard deviation; SEM, standard error of the mean.

We analyzed plasma NfL, beta-synuclein, and GFAP levels at four different time points (0 h, 24 h, 5 d, and 10 d after hospital admission) in 32 patients after severe injury and in an additional cohort of 13 healthy volunteers (Table 2). Plasma NfL steadily increased over time, with significantly higher concentrations compared to controls from 24 h onward (*p* < 0.0001) and the highest concentrations after 10 d (Figure 1A). In contrast, plasma beta-synuclein concentrations were already significantly higher at 0 h compared to the control group (*p* < 0.0001) (Figure 1B). The synaptic marker continually decreased in the time course after trauma but still showed significantly higher levels at 10 d compared to the control group (*p* = 0.03). Plasma GFAP also displayed significantly higher concentrations at 0 h compared to the healthy volunteers, but its concentration declined over time, with levels in the range of the control group after 10 d (*p* = 0.5) (Figure 1C). Marker concentrations in direct comparison are shown in Figure 1D.

### 2.3. Correlations of Neurochemical Markers with Clinical Scores and Markers Assessed in the Emergency Room

**Figure 2:**
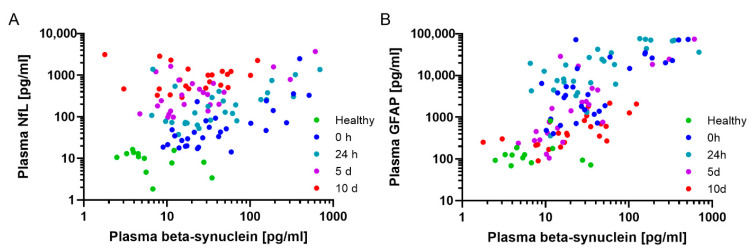

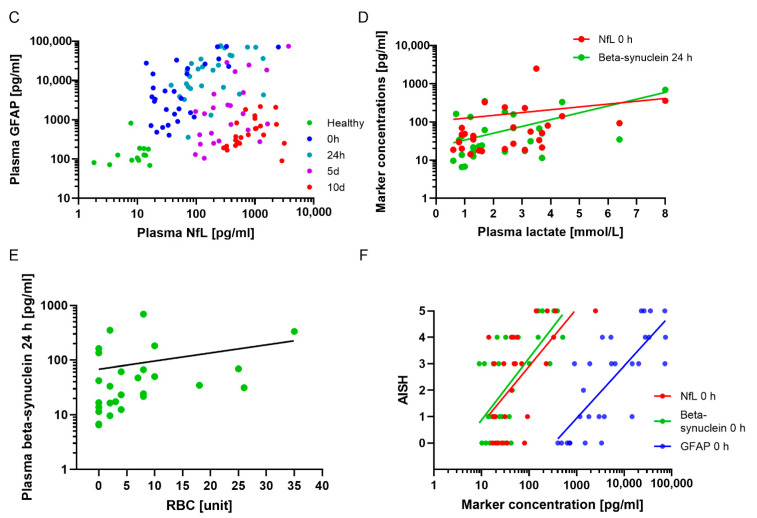
Correlations between markers and clinical scores. (**A**–**C**) Correlations between neurological markers in the trauma cohort; healthy controls are shown as a reference. Beta-synuclein and NfL (**A**) (r = 0.16 (CI: −0.04–0.35), *p* = 0.1) and GFAP (**B**) (r = 0.63 (CI: 0.50–0.74), *p* < 0.0001) as well as NFL and GFAP (**C**) (r = −0.10 (CI: 0.29–0.10), *p* = 0.3), with color coded time points, respectively. Individual r and *p* values for the different times can be found in Supplementary Table S1. N = 117 for (**A**–**C**). (**D**) Correlation between plasma lactate assessed in the shock room and NfL (r = 0.47 (CI: 0.10–0.72), n = 28, *p* = 0.01) and beta-synuclein (r = 0.40 (CI: 0.01–0.68), n = 27, *p* = 0.03) at 0 h and 24 h, respectively. (**E**) Correlation between the applied RBC units and plasma beta-synuclein at 24 h (r = 0.45 (CI: 0.07–0.71), n = 27, *p* = 0.02). (**F**) Correlation analysis of the AISH with all three neurological plasma markers 0 h postadmission (NfL (r = 0.56 (CI: 0.24–0.77), n = 29, *p* = 0.0015), beta-synuclein (r = 0.63 (CI: 0.33–0.81), n = 29, *p* = 0.0003), and GFAP (r = 0.80 (CI: 0.61–0.91), n = 29, *p* < 0.0001)). Individual r and *p* values of the AISH with other time points can be found in Supplementary Table S1. Correlation analysis was performed using Spearman’s rank correlation coefficient. Lines represent nonlinear regression. AISH, abbreviated injury score head; GFAP, glial fibrillary acidic protein; NfL, neurofilament light chain; RBC, red blood cell concentrates.

A correlation analysis (Figure 2A–C) revealed no correlation between NfL and beta-synuclein (Figure 2A) as well as NfL and GFAP (Figure 2C), but there was a significant association between beta-synuclein and GFAP in the trauma cohort when assessing all time points postadmission (Figure 2B, r = 0.63 (CI: 0.50–0.74), *p* < 0.0001). At the single time points, we observed a moderate to strong correlation between beta-synuclein and NfL at 0 h, 24 h, and 5 d after admission as well as a moderate to strong correlations with GFAP at all time points after injury. NfL and GFAP displayed weak to moderate correlations at all analyzed time points (see Supplementary Table S1 for specific values). Furthermore, we found no correlation between the markers in the healthy volunteer group. A correlation analysis between the clinical parameters (initial interleukin (IL)-6, initial base excess, initial lactate, and red blood cell concentrates (RBC) administered during the first 24 h) revealed no association to any of the neurochemical markers at all time points, with the notable exceptions of NfL and beta-synuclein with lactate levels at 0 h and 24 h, respectively (NfL 0 h: *r* = 0.47 (CI: 0.10–0.72), *p* = 0.01, beta-synuclein 24 h: *r* = 0.40 (CI: 0.01–0.68), *p* = 0.03) (Figure 2D), and beta-synuclein and RBC given during the first 24 h (*r* = 0.45 (CI: 0.07–0.71), *p* = 0.02) (Figure 2E). Furthermore, we observed no correlation with overall injury severity (ISS) but a strong correlation of the abbreviated injury scale of the head (AISH) with NfL (*r* = 0.56 (CI: 0.24–0.77), *p* = 0.0015), beta-synuclein (*r* = 0.63 (CI: 0.33–0.81), *p* = 0.0003), and GFAP (*r* = 0.80 (CI: 0.61–0.91), *p* < 0.0001) at time point 0 h (Figure 2F) (see Supplementary Table S1 for correlations with other time points).

### 2.4. Discrimination of TBI vs. Non-TBI

**Figure 3:**
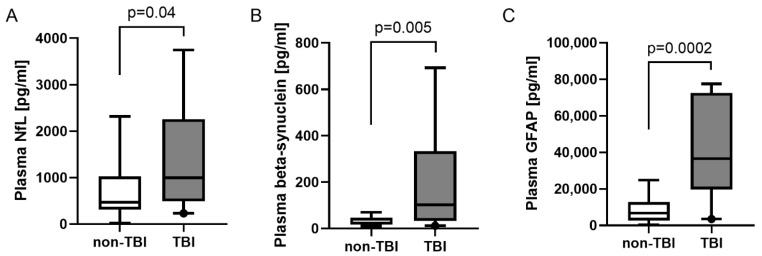
Differences and discrimination between severe trauma patients with and without TBI. (**A**–**C**) Difference in maximum observed plasma concentrations of NfL (**A**), beta-synuclein (**B**), and GFAP (**C**) between severely injured trauma patients with and without TBI. Concentrations are displayed as box plots. The median concentration, the 25% and 75% percentiles, and whiskers from 5% to 95% are illustrated. Groups were compared by Mann–Whitney U test. N = 19 (with TBI), and n = 13 (without TBI). GFAP, glial fibrillary acidic protein; NfL, neurofilament light chain; TBI, traumatic brain injury.

To analyze the neurochemical triplet in relation to brain injury, we stratified our patient cohort into patients with and without TBI. For the discrimination of non-TBI vs. TBI, the maximum observed levels of NfL, beta-synuclein, and GFAP per patient over the time course post-trauma were significantly higher in the TBI group compared to non-TBI patients (Figure 3A–C). A power analysis demonstrated that, with the observed effect sizes, 28 patients per group would be sufficient to detect significant differences between TBI and non-TBI polytrauma patients in all three neuronal marker analyses in future studies. For beta-synuclein and GFAP alone, the required patient numbers per group were found to be nine and six patients, respectively.

### 2.5. Marker Levels over Time in Severe Trauma Patients with and without TBI

**Figure 4:**
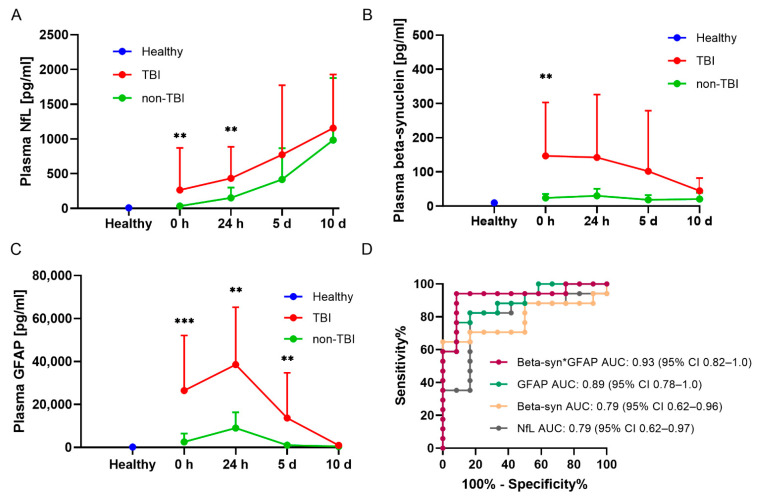
Marker concentrations in the time course of injury in multiple trauma with and without TBI. (**A**–**C**) Plasma (NfL) (**A**), beta-synuclein (**B**), and GFAP (**C**) concentrations in the time course of polytrauma with and without TBI compared to the levels of healthy volunteers. The mean plasma concentrations + SD are displayed for the individual sampling dates. Differences in marker levels between TBI and non-TBI at single time points were assessed with Mann–Whitney U test. *** *p* < 0.001, ** *p* < 0.01 vs. non-TBI. N(healthy) = 13; 0 h: N(TBI) = 17, n(non-TBI) = 12; 24 h: N(TBI) = 17, n(non-TBI) = 11; 5 d: N(TBI) = 14, n(non-TBI) = 10; 10 d: N(TBI) = 12, n(non-TBI) = 11. (**D**) Comparison of ROC curve analysis for the discrimination between TBI and non-TBI trauma patients for plasma NfL, beta-synuclein, GFAP, and the product of beta-synuclein and GFAP at admission (0 h) to the clinic. (For 0 h: TBI n = 17, non-TBI n = 12). AUC, area under the curve; GFAP, glial fibrillary acidic protein; NfL, neurofilament light chain; ROC; receiver operating characteristic; SEM, standard error of the mean; TBI, traumatic brain injury.

In the time course of injury, NfL, beta-synuclein, and GFAP showed higher levels in the TBI group compared to patients who did not sustain TBI. Plasma NfL concentrations were significantly elevated after 0 and 24 h in TBI compared to non-TBI patients (*p* = 0.007 and *p* = 0.003, respectively) but displayed no significant differences after 5 and 10 d (Figure 4A). In comparison to the healthy control group, NfL levels were clearly elevated from 24 h onwards in the TBI and non-TBI group. Beta-synuclein plasma concentrations trended higher after TBI than at corresponding time points in the non-TBI group (Figure 4B). Significantly elevated concentrations were found after 0 h (*p* = 0.008). In comparison to healthy controls, TBI patients showed increased levels at all time points, while the beta-synuclein concentrations in the non-TBI group were relatively similar to those measured in the healthy volunteers. For GFAP concentrations, we found significantly higher levels after 0 h (*p* = 0.0002), 24 h (*p* = 0.004), and 5 d (*p* = 0.009) compared to the corresponding time points in the non-TBI group, with assimilating levels after 10 d (Figure 4C). Compared to the control group, GFAP concentrations in the TBI group displayed elevated levels at all time points except 10 d, whereas there was a peak in the non-TBI group only 24 h postadmission. The biomarker concentrations in the TBI group after stratification according to AISH 2–3 and 4–5 are shown in Supplementary Figure S1. In addition, an ROC curve analysis of non-TBI vs. TBI demonstrated a good discriminating potential within the polytrauma setting at the initial blood draw for all three markers and especially for the combination of beta-synuclein and GFAP (Figure 4D). The receiver operating characteristic (ROC) analysis revealed areas under the curve (AUCs) of 0.90 (NfL*GFAP), 0.89 (NfL*beta-synuclein*GFAP), and 0.85 (NfL*beta-synuclein) for the other combinations. The corresponding curves and 95% CIs can be found in Supplementary Figure S2.

### 2.6. Marker Concentrations in Survivors and Nonsurvivors

**Figure 5:**
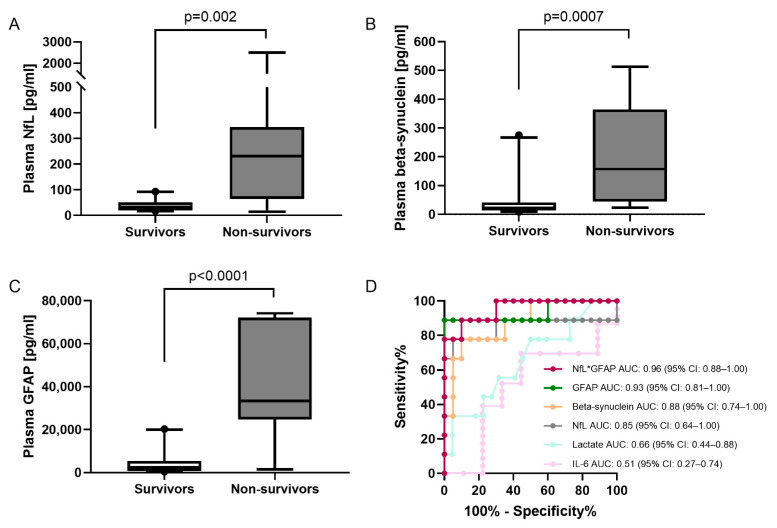
Differences and discrimination between survivors and nonsurvivors. (**A**–**C**) Differences in initial (0 h) plasma concentrations of NfL (**A**), beta-synuclein (**B**), and GFAP (**C**) between survivors and nonsurvivors. Concentrations are displayed as box plots. The median concentration, the 25% and 75% percentiles, and whiskers from 5% to 95% from n = 20 survivors and n = 9 nonsurvivors are illustrated. Groups were compared by Mann–Whitney U test. (**D**) Comparison of ROC curve analysis for the discrimination between survivors and nonsurvivors for plasma NfL, beta-synuclein, GFAP, lactate, IL-6, and the product of NfL and GFAP. AUC, area under the curve; GFAP, glial fibrillary acidic protein; NfL, neurofilament light chain; ROC, receiver operating characteristic.

To evaluate the three markers in regard to outcome, we stratified the trauma patient cohort into patients who survived their injuries and patients who did not. Plasma NfL, beta-synuclein, and GFAP displayed significantly higher levels at the initial time point 0 h in patients who did not survive their injuries (Figure 5A–C). Furthermore, ROC curve analysis demonstrated higher AUCs for all three biomarkers for discrimination between survivors and nonsurvivors at 0 h compared to lactate and IL-6, which are routinely assessed in the emergency room. This was especially true for the combination of NfL and GFAP (Figure 5D). AUCs of the other combinations were 0.94 (NfL*beta-synuclein*GFAP), 0.92 (NfL*beta-synuclein), and 0.90 (GFAP*beta-synuclein). The corresponding curves and 95% CIs can be found in Supplementary Figure S3. Moreover, we selected the cut-off with the highest likelihood ratio for nonsurvival, demonstrating that patients with one of the markers above the respective cut off at the time of admission (NfL > 77 pg/mL, beta-synuclein > 129 pg/mL, and GFAP > 17,553 pg/mL) had a 15-, 13-, or 17-times higher chance to die of their injury, respectively. In those patients who survived to hospital discharge, there was no correlation between the initial neuromarker plasma concentrations and the length of stay on the intensive care unit or in the hospital.

## 3. Discussion

Here, we report on the analysis of the three neurological markers NfL, beta-synuclein, and GFAP in the time course after severe trauma with the first overall blood measurement of a synaptic protein in trauma patients. Our study extends the principal understanding of neurofilaments and GFAP in TBI and especially in patients who sustained severe trauma without primary brain injury.

For NfL concentrations, we observed a gradual increase in the trauma cohort over the 10 days of clinical monitoring. To the best of our knowledge, there are so far no other studies investigating NfL in the time course after severe trauma. However, studies investigating NfL in patients with isolated TBI have seen the same steady rise in NfL over time postinjury [14,19], with an initial increase, presumably due to early brain injury and concomitant neuronal destruction. In line with our findings, neurofilament immunoreactivity in the injured region was found to be strongly decreased in brain tissue until 7 days after TBI [20], suggesting a breakdown of the neuronal cytoskeleton and the subsequent release of structural proteins such as neurofilaments into the systemic circulation. The gradual increase in our cohort, with the highest levels after 10 days, even in non-TBI patients, however, may be caused by secondary effects after injury leading to delayed neuroaxonal degeneration, e.g., by iron toxicity, oxidative stress, lagged ischemia, and/or general inflammation [21,22,23].

In contrast, the plasma concentrations of brain-specific beta-synuclein, reflecting synaptic damage [8,24,25,26], already peaked early after injury. Here, the immediate increase was most likely due to early brain damage leading to the demise of synapses. This is supported by the fact that the initial plasma beta-synuclein levels were related to the severity of the traumatic hit, and delayed secondary factors appeared to have smaller effects on synapse stability. Our data also indicate that synapse degeneration in this patient cohort gradually subsided from 24 h after hospital admission onwards. GFAP, an intermediate filament protein in the astrocyte cytoskeleton, is released into the CSF/blood after neurotrauma [27]; hence, plasma GFAP can be considered an extensively studied and well-established surrogate marker of astroglial activation or damage in TBI [16,27,28]. GFAP immunostaining in brain tissues explanted after different time periods after closed head injury revealed a high number of patients with positive immunostaining, especially during the first days post-trauma [29]. We corroborated the data from these studies with our findings, observing an increase in GFAP blood concentrations directly after trauma, with a peak at 24 h, followed by a gradual decline [30].

Possibly due to the similar time course of plasma levels for beta-synuclein and GFAP, correlation analyses revealed a significant association between the two markers in the whole trauma cohort and at all single time points. In contrast, there was no significant association between beta-synuclein and NfL when assessing all time points collectively. However, we found significant associations when analyzing early time points separately, underlining the difference in the two markers, especially during the later course after injury. Interestingly, we observed correlations between NfL, beta-synuclein, and blood lactate as well as between beta-synuclein and erythrocyte concentrates given during the first day at early time points postinjury. As an end product of anaerobic metabolism, high plasma lactate concentrations in trauma may reflect cellular hypoxia and are typically observed in patients after hemorrhagic shock [31,32]. Furthermore, severely injured patients are transfused with RBCs to maintain or re-establish optimal oxygen-carrying capacity. Consequently, patients with high lactate and transfusion demand most likely also suffered from the highest extent of ischemia and hypoxia in the brain during the initial injury phase, leading to neuronal cell death and the release of NfL and beta-synuclein into the extracellular space and subsequently the blood stream. Interestingly, astrocytes are much more resistant to hypoxia than neurons [33], which was reflected in the absent correlation between GFAP and lactate concentrations or RBC numbers in our cohort. As can be expected for brain-related markers, beta-synuclein, NfL, and GFAP displayed strong associations with the severity of head injury. This prompted us to stratify our cohort into patients with and without TBI. At the time of admission, all three markers differentiated well between trauma patients who sustained a TBI and those who did not, with the combination of GFAP and beta-synuclein showing superior discriminating potential (AUC: 0.93) compared to the markers alone (GFAP AUC: 0.89, beta-synuclein AUC: 0.79, and NfL AUC: 0.79). This finding promotes the idea of combining neurological markers reflecting structurally different injuries to the brain to obtain a fluid-biomarker-supported TBI diagnosis, which has to be confirmed in future studies in a larger patient cohort. To the best of our knowledge, this is the first study assessing the discriminating potential of GFAP, NfL, and the novel synaptic injury marker beta-synuclein using a digital ELISA-based test in plasma of severe trauma patients with and without TBI. Our results are in line with a study on serum GFAP where discrimination between isolated TBI and non-TBI trauma patients revealed an AUC for GFAP of 0.89 [34]. Furthermore, a recent study comparing NfL and GFAP in rugby players with and without mild TBI also observed superior results for GFAP [35]. In addition, the relatively small patient numbers required for potential future studies in polytrauma patients, as estimated by the power analysis based on our observed effect sizes, indicate a high validity of the results in the present cohort. However, despite the effective discriminating potential of all three markers, the plasma concentrations strongly differed in the time course of TBI and especially in the non-TBI patients. An explanation might be found in the expression profiles of the three proteins. Beta-synuclein, expressed nearly exclusively in the brain [25,26], is scarcely released in trauma patients without primary head injury. GFAP is brain-enriched but is also expressed in peripheral nonmyelinating Schwann cells and in enteric glia cells, albeit to a much lower extent [36,37]. The increase in plasma GFAP after 24 h in the non-TBI group might therefore be due to gut–blood barrier dysfunction and glial cell activation in the intestine. In contrast, NfL levels continually increased postinjury in the non-TBI cohort, which was conceivably caused by the degeneration of neurons in the peripheral nervous system, where NfL is substantially expressed in axons [38,39]. In this regard, it has been shown that patients with Charcot–Marie–Tooth disease, a group of inherited disorders that cause peripheral nerve damage, as well as patients with an acquired peripheral neuropathy, have elevated NfL levels compared to control patients [40,41].

Regarding survival after trauma, our findings for beta-synuclein, NfL, and GFAP plasma concentrations upon admission demonstrate that all three markers are able to predict mortality in severe trauma patients at the earliest possible time point. So far, this has only been shown for NfL and GFAP alone at later time points postinjury [14,34]. Moreover, in our cohort, the combination of markers further increased the predictive power regarding fatal outcomes. Furthermore, the neurological markers outperformed laboratory markers that are routinely determined upon admission, such as blood lactate and IL-6, in the prediction of mortality. In addition, we were the first to observe the predictive power of a synaptic protein, namely, beta-synuclein, in the plasma of trauma patients concerning mortality. However, future validation studies in larger patient cohorts have yet to confirm the meaningfulness of our findings.

The strengths of the present study are (i) the cohort of severely injured patients with and without TBI, mirroring the patient collective seen in clinical emergency rooms with a large need for highly effective clinical treatment strategies and reliable biomarkers; (ii) it is the first analysis of a synaptic biomarker in the blood of trauma patients; (iii) the comparison of three biomarkers, representing different neurological aspects, in TBI and non-TBI and the correlation with each other and central clinical parameters, (iv) the reliable detection of TBI within a severely injured patient cohort despite the “noise” of numerous released damage–inflammation mediators; and (v) the assessment of the predictive potential of all markers at the earliest possible time point concerning survival. The study might be limited by (i) the short follow-up time of 10 days, which prevented us from a closer monitoring of brain-related outcomes, and (ii) the relatively small number of patients, hindering further stratification.

In this study, we present the first longitudinal study on severe trauma patients including a plasma synaptic biomarker, demonstrating elevated levels of beta-synuclein, NfL, and GFAP in the time course after injury, with a high early predictive potential of outcomes. Moreover, we observed correlations to central clinical parameters and substantially differing biomarker courses for beta-synuclein, NfL, and GFAP in TBI and non-TBI patients. Early monitoring of neuronal damage employing established and novel plasma biomarkers may support the fast diagnosis and monitoring of TBI in clinical settings without the option of imaging technology and thus may aid in more effective treatment strategies for the critically injured.

## 4. Materials and Methods

### 4.1. Patients

We performed a prospective, monocentric, longitudinal, controlled, observational study in polytraumatized patients after approval from the local ethics committee. The samples examined in this study were taken from n = 32 consecutive patients with severe injury that were admitted to the emergency room of a level I trauma center at Ulm University Hospital between 2013 and 2016. All patients underwent clinical examinations and neuroimaging and were included if their injury severity score (ISS), calculated after whole-body computed tomography, was 25 or higher. The exclusion criteria were age < 18 years, pregnancy, infection with the human immunodeficiency virus, cardiogenic shock as the primary underlying disease, underlying hematologic disease, cytotoxic therapy within the previous 6 months, and the presence of rapidly progressing underlying disease anticipating death within the next 24 h. Patients were only enrolled if the time between the traumatic injury and their arrival at the emergency department was less than 4 h. Venous blood was drawn at the time of admission to the emergency room (0 h) and 24 h, 5 d, and 10 d later, with a maximum divergence of ±10%. N = 13 healthy volunteers served as a control group. Clinical data, including initial IL-6, initial base excess, initial lactate, and red blood cell concentrates (RBC) administered during the first 24 h, were collected retrospectively from the patients’ files. Eight patients did not survive the first six days after admission to the hospital. One patient never regained full consciousness after admission and died 77 days later.

**Figure 6:**
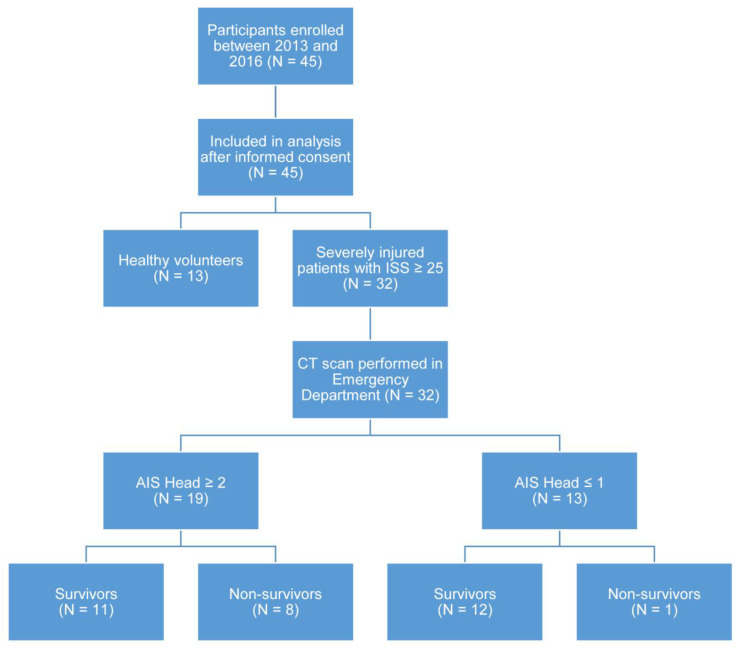
Flow chart of enrolled participants. AIS, abbreviated injury score; CT, computed tomography; ISS, injury severity score.

For the stratification of the patient cohort into TBI and non-TBI, the abbreviated injury scale of the head (AISH) was determined on an AIS-2015-based evaluation of the polytrauma CT-scan performed on admission to the hospital [42]. The AIS was used since the Glasgow coma score can be limited in the setting of polytrauma due to intubation/ventilation, morphine/sedatives, and narcotics. Patients with an AISH ≥ 2 were classified as patients who sustained a moderate–severe TBI [43,44]. Participant enrollment is shown in Figure 6.

### 4.2. Plasma Collection and Analysis

Whole blood was drawn into tubes containing ethylenediaminetetraacetic acid as anticoagulant (Sarstedt, Nümbrecht, Germany). Plasma was extracted from blood samples by centrifugation (2000× *g*, 10 min), and aliquots were immediately stored at −80 °C until analysis. NfL, beta-synuclein, and GFAP were retrospectively analyzed in the plasma samples of patients and healthy volunteers.

Plasma NfL was quantified using a commercially available kit for the microfluidic ELLA system (Bio-Techne, Minneapolis, MN, USA). Plasma GFAP was analyzed with the commercially available Discovery single-molecule array (Simoa) kit for the HD-1 platform (Quanterix, Billerica, MA, USA) according to the manufacturers’ instructions. Beta-synuclein plasma levels were measured employing a novel in-house Simoa assay using the HD-1 platform (please see the Supplementary Materials for detailed method and validation data).

### 4.3. Statistical Analysis

Normality of distribution was assessed employing the Shapiro–Wilk test. The Kruskal–Wallis test with subsequent Dunn´s post hoc correction for multiple testing was used to analyze differences between three or more groups. Two groups were compared with the Mann–Whitney U test or chi-squared testing for sex differences. Receiver operating characteristic (ROC) analyses were applied for the determination of cut-offs, with an area under the curve (AUC) of 1 indicating a diagnostic sensitivity and specificity of 100 % and an AUC of 0.5 suggesting no discrimination between diagnostic groups. For associations between markers and clinical scores and parameters, Spearman rank correlation was performed. For all analyses, *p* < 0.05 was considered statistically significant. A power analysis for the discrimination between polytrauma patients with and without TBI employing the Mann–Whitney U test was performed with a predefined power of 80%, an alpha level of 5%, and the observed effect sizes in the present analyses. Statistical analysis was performed with GraphPad Prism 8.0 software (GraphPad Software, La Jolla, CA, USA).

## 5. Patents

The foundation of the state Baden-Wuerttemberg handed in a patent for the measurement of beta-synuclein.

## Figures and Tables

**Table 1:** Demographic and clinical features of patients and healthy volunteers.

	Healthy	Polytrauma All		TBI	Non-TBI	
N	13	32		19	13	
m/f	8/5	26/6	*p* = 0.2	17/2	9/4	*p* = 0.2
Age (y)	32 (26–49.5)	47.5 (34.8–58)	*p* = 0.06	46 (37–52)	53 (30.5–58.5)	*p* = 0.5
ISS	n/a	34 (29–41)		34 (29–43)	34 (29–39.5)	*p* = 0.5
AISH	n/a	3 (0.3–4)		4 (3–4)	0 (0–1)	*p* < 0.0001
Initial base excess [mmol/l]	n/a	−2.9 (−5.3–−1.7)		−2.4 (−4.6–−0.9)	−3.1 (−5.4–−2.7)	*p =* 0.3
Initial lactate [mmol/l]	n/a	2.4 (1.2–3.6)		1.7 (0.9–3.3)	2.9 (1.5–3.7)	*p =* 0.1
RBC in first 24 h [units]	n/a	3 (0–8)		2 (0–10)	4 (0–8)	*p =* 0.8
Initial IL-6 [pg/mL]	n/a	235 (84–827)		123 (38–478)	534 (216–969)	*p =* 0.07

Parameters are shown as median (IQR). AISH, abbreviated injury scale head; IL-6, interleukin-6; IQR, interquartile range; ISS, injury severity score; RBC, red blood cell concentrates; TBI, traumatic brain injury.

**Table 2:** Plasma marker concentrations.

	Healthy	0 h	24 h	5 d	10 d
Analyzed samples	n = 13	n = 29	n = 28	n = 24	n = 23
NfL [pg/mL] (mean (SD); median (IQR))	9.9 (4.6); 10.6 (6.2–13.5)	163 (459); 43.1 (20.8–86.6)	314 (379); 154 (74.6–360)	611 (802); 340 (150–734)	1067 (824); 774 (472–1246)
Beta-synuclein [pg/mL] (mean (SD); median (IQR))	9.8 (10); 5.3 (3.9–11.8)	92 (130); 29.4 (17.1–129)	94 (149); 34.1 (16.6–119)	64 (135); 18.2 (10.7–35.5)	32 (31); 18.2 (10.9–46.2)
GFAP [pg/mL] (mean (SD); median (IQR))	174 (198); 1 25 (87.2–182)	15,768 (22,438); 3832 (1287–24,745)	25,887 (25,297); 18,225 (6361–41,277)	7892 (16,551); 1428 (319–4838)	672 (617); 412 (251–833)

For patients, the numbers of analyzed samples differing from 32 are due to missing samples or death before the end of the 10-day observation period. GFAP, glial fibrillary acidic protein; IQR, interquartile range; NfL, neurofilament light chain; SD, standard deviation.

## Data Availability

The data presented in this study are available on request from the corresponding author. The data are not publicly available due to ethical concerns.

## Supplementary Materials

The supporting information can be downloaded at: https://www.mdpi.com/article/10.3390/ijms23179639/s1.
