# Supplementary material for: Neurochemical Monitoring of Traumatic Brain Injury by the Combined Analysis of Plasma Beta-Synuclein, NfL, and GFAP in Polytraumatized Patients
Source: Int J Mol Sci. 2022 Aug 25;23(17):9639. doi: 10.3390/ijms23179639 (PMC9456193; doi:10.3390/ijms23179639)
Supplement: Supplementary file 1 [file ijms-23-09639-s001.zip › ijms-1835674-supplementary.pdf]

# Neurochemical Monitoring of Traumatic Brain Injury by the Combined Analysis of Plasma Beta-Synuclein, NfL, and GFAP in Polytraumatized Patients

## Supplementary materials

### Beta-synuclein Simoa assay

A specific beta-synuclein capture antibody (EP1537Y, Abcam, Cambridge) was coupled to carboxylated paramagnetic beads (Quanterix, MA, USA) with a coating concentration of 0.2 mg/ml using the manufacturer's protocol. 125.000 beads and 375.000 helper beads (Quanterix, MA, USA) were applied per replicate. The biotinylated detection antibody recognizing alpha- and beta-synuclein (EP1646Y, Abcam, Cambridge) was used in a concentration of 0.5 µg/ml with a biotin to antibody ratio of 40:1. PBS-Tween 0.5% and PBS-Tween 0.05% were used as bead and detector diluent, respectively. A concentration of 150 pM Streptavidin-β-galactosidase (SBG) buffer provided by Quanterix was applied. As substrate, Resorufin β-D-Galactopyranoside (RGP) (Quanterix, MA, USA) was used. Recombinant beta-synuclein protein (rPeptide, Watkinsville, GA, USA) was purchased to prepare calibrators ranging from 0.625 to 100 pg/ml (the exact beta-synuclein concentration of the stock solution was quantified by amino acid analysis (Alphalyse A/S, Odense, Denmark). 400 µl of each calibrator was added to a 96-well plate provided by Quanterix. Plasma samples were diluted 1:8 and 220 µl of each sample was added to the plate. After placing beads, detector, SBG, plate, and substrate into the HD-1 platform, a 3-step custom assay was applied for measurement.

No cross-reaction with alpha- and gamma-synuclein was observed. The intra- and interassay CV was determined to be 4% and 11%, respectively. By spike-in experiments, a mean recovery rate of 98% was measured. For this assay, the lower limit of quantification was calculated to be 1.0 pg/ml. Dilutions between 1:2 and 1:8 showed dilution stability for blood plasma samples.

## Correlation analysis

**Supplementary Table S1: Correlation of healthy patients and single time points after trauma between the three markers as well as the AISH**

| Correlation | Healthy | 0 h | 24 h | 5 d | 10 d |
|---|---|---|---|---|---|
| **Beta-synuclein and NfL** | r=-0.55 (-0.85-0.02), p=0.06 | r=0.66 (0.38-0.83), p<0.0001 | r=0.51 (0.16-0.75), p=0.0052 | r=0.46 (0.07-0.74), p=0.02 | r=0.24 (-0.20-0.61), p=0.26 |
| **Beta-synuclein and GFAP** | r=0.01 (-0.55-0.57), p=0.98 | r=0.62 (0.31-0.81), p=0.0004 | r=0.70 (0.43-0.85), p<0.0001 | r=0.77 (0.52-0.90), p<0.0001 | r=0.70 (0.39-0.87), p=0.0002 |
| **NfL and GFAP** | r=0.17 (-0.44-0.66), p=0.58 | r=0.44 (0.08-0.70), p=0.016 | r=0.49 (0.13-0.73), p=0.0089 | r=0.53 (0.15-0.77), p=0.0078 | r=0.43 (0.002-0.72), p=0.04 |
| **AISH and Beta-synuclein** | N/A | r=0.56 (0.24-0.77), p=0.0015 | r=0.58 (0.26-0.79), p=0.0011 | r=0.48 (0.08-0.74), p=0.019 | r=0.36 (-0.08-0.68), p=0.09 |
| **AISH and NfL** | N/A | r=0.63 (0.33-0.81), p=0.0003 | r=0.48 (0.12-0.73), p=0.009 | r=0.42 (0.004-0.71), p=0.04 | r=0.42 (-0.003-0.72), p=0.04 |
| **AISH and GFAP** | N/A | r=0.80 (0.61-0.91), p<0.0001 | r=0.64 (0.34-0.82), p=0.0002 | r=0.64 (0.31-0.83), p=0.0007 | r=0.49 (0.08-0.75), p=0.02 |

Shown is the spearman correlation r with 95% confidence interval in parentheses. Orange color indicates a significant correlation. AISH, abbreviated injury score of the head; d, days; GFAP, Glial fibrillary acidic protein; h, hours; N/A, not applicable; NfL, neurofilament light chain.

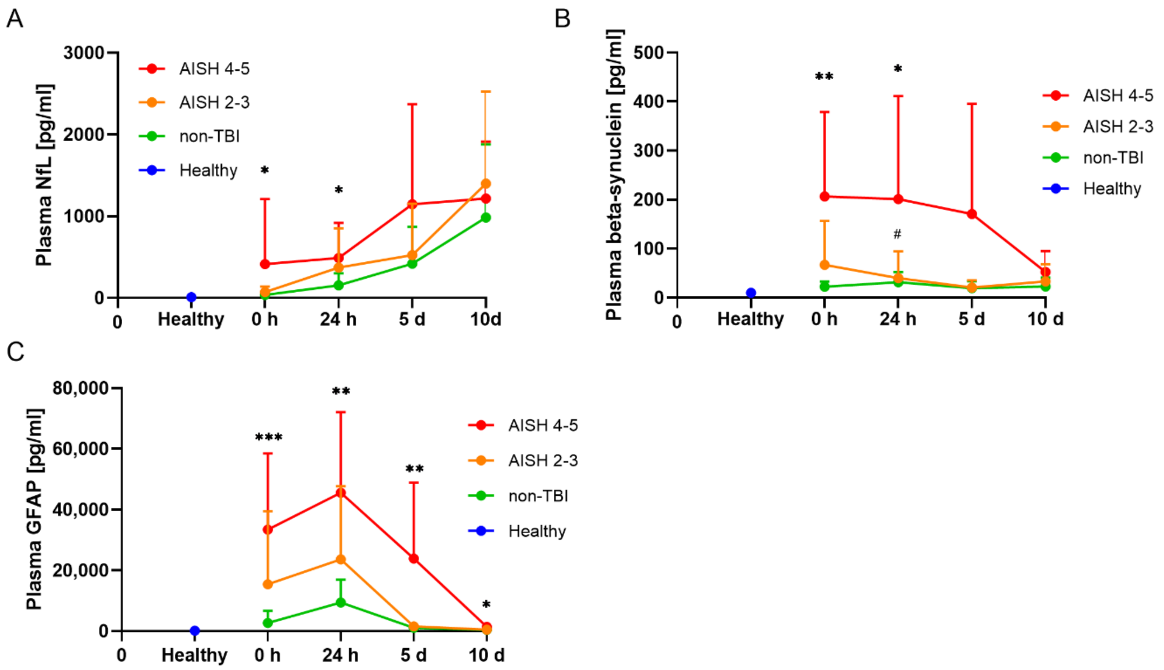


**Supplementary Figure S1: Marker concentrations in the time course of injury in severe trauma with TBI stratified according to AISH 2-3 and 4-5 and without TBI**

(A-C) Plasma (NfL) (A), beta-synuclein (B) and GFAP (C) concentrations in the time course of severe trauma with TBI 2-3 and 4-5 and without TBI compared to levels of healthy volunteers. Displayed is the mean plasma concentration + SD for the individual sampling dates. Differences in marker levels between TBI 2-3 and TBI 4-5 as well as non-TBI at single time points were assessed with Kruskal-Wallis test with subsequent Dunn's post hoc test. *** $p<0.001$, ** $p<0.01$, * $p<0.05$ vs. non-TBI. # $p<0.05$ TBI 2-3 vs. TBI 4-5. GFAP, glial fibrillary acidic protein; NfL, neurofilament light chain; SEM, standard error of the mean; TBI, traumatic brain injury

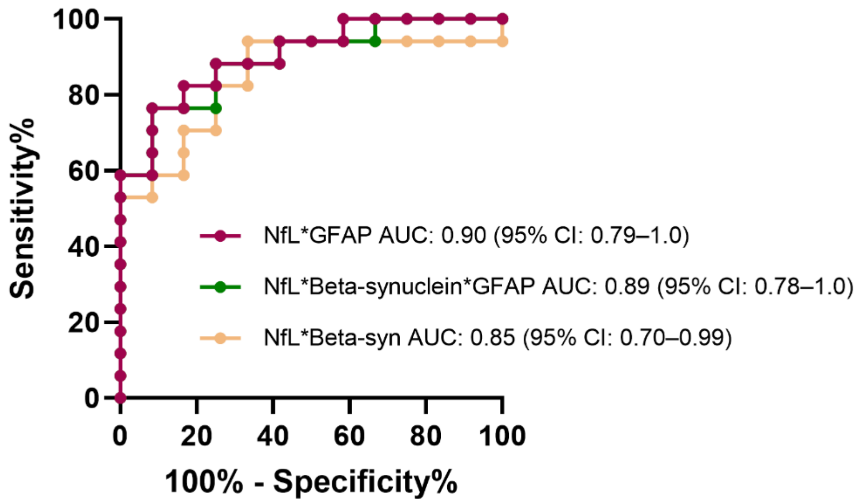


**Supplementary Figure S2: Additional ROC curves for the discrimination between TBI and non-TBI trauma patients**

Shown are the ROC curves from the plasma level products at 0 h of NfL and GFAP, NfL and beta-synuclein as well as NfL and beta-synuclein and GFAP. GFAP, glial fibrillary acidic protein; NfL, neurofilament light chain; ROC, receiver operating characteristics; TBI, traumatic brain injury.

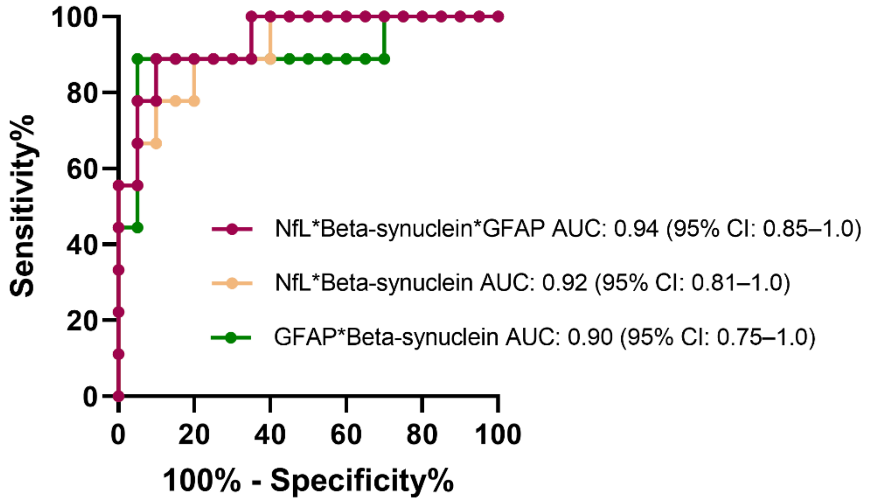


**Supplementary Figure S3: Additional ROC curves for the discrimination between survivors and non-survivors**

Shown are the ROC curves from the plasma level products at 0 h of NfL and Beta-synuclein, GFAP and beta-synuclein as well as NfL and beta-synuclein and GFAP. GFAP, glial fibrillary acidic protein; NfL, neurofilament light chain; ROC, receiver operating characteristics.
